# Offspring fitness varies with parental extra-pair status in song sparrows, *Melospiza melodia*

**DOI:** 10.1098/rspb.2012.1139

**Published:** 2012-08-08

**Authors:** Rebecca J. Sardell, Peter Arcese, Jane M. Reid

**Affiliations:** 1 Institute of Biological and Environmental Sciences, School of Biological Sciences, Zoology Building, University of Aberdeen, Tillydrone Avenue, Aberdeen AB24 2TZ, UK; 2 Department of Biological Science, Florida State University, 319 Stadium Drive, Tallahassee, FL 32306-4295, USA; 3 Centre for Applied Conservation Research, Department of Forest Sciences, University of British Columbia, 2424 Main Mall, Vancouver, British Columbia, CanadaV6T 1Z4

**Keywords:** extra-pair paternity, fitness, half-sibling, inter-generational maternal effects, polyandry

## Abstract

Numerous studies have tested for indirect selection on female extra-pair reproduction (EPR) by quantifying whether extra-pair young (EPY) are fitter than their within-pair young (WPY) maternal half-siblings. In contrast, the hypothesis that offspring of EPY and WPY (rather than the EPY and WPY themselves) differ in fitness has not been tested, even though inter-generational effects of parental extra-pair status on offspring fitness could alter the magnitude and direction of indirect selection on EPR. We tested whether offspring of EPY song sparrows, *Melospiza melodia*, were more likely to recruit or produce hatched or recruited offspring over their lifetimes than offspring of WPY. Hatchlings with one or two EPY parents were more likely to recruit and produce hatched offspring than hatchlings with two WPY parents. Furthermore, these relationships differed between maternal versus paternal extra-pair status. Hatchlings with EPY fathers were more likely to recruit and produce offspring than hatchlings with WPY fathers. In contrast, hatchlings with EPY mothers were as likely to recruit as hatchlings with WPY mothers and tended to be less likely to produce recruited offspring. Depending on the causal genetic and environmental mechanisms, such conflicting inter-generational relationships between parental extra-pair status and offspring fitness could substantially influence the evolutionary dynamics of EPR.

## 1. Introduction

Understanding the evolutionary forces that drive extra-pair reproduction (EPR) in socially monogamous species ultimately requires that all components of direct and indirect selection acting on females and males be quantified [1–5]. One key force driving the evolution of female EPR, and hence polyandry, is hypothesized to be positive indirect selection resulting from increased fitness of extra-pair offspring [1,6]. This hypothesis predicts that extra-pair young (EPY) will be fitter than their within-pair young (WPY) maternal half-siblings from the same brood or litter, potentially reflecting a difference in paternal genetic contribution [1,6–8] (although see [9–11]). Numerous studies have tested this prediction by comparing morphological, physiological and life-history phenotypes between maternal half-sibling EPY and WPY [1,7,8,12,13] (see the electronic supplementary material, figure S1).

However, in general, phenotypic variation may be correlated across generations via various mechanisms [14–20]. This is by definition true for heritable traits that show additive genetic variation [15,21,22]. Parents and offspring may also resemble each other in heterozygosity and inbreeding coefficient (*f*), and hence in traits that show inbreeding depression [23–27]. Furthermore, inter-generational phenotypic effects of current or previous environments have been demonstrated in a wide range of contexts and systems, including effects of environment per se, and non-genetic maternal and paternal effects [28–33].

Such inter-generational relationships could cause phenotypic effects of extra-pair status to be manifested across generations. Specifically, if EPY and WPY differ in additive genetic value (as widely hypothesized [1,6]), they will produce offspring that also differ in additive genetic value (and hence phenotype), on average. Inter-generational relationships between parental extra-pair status and offspring fitness could also arise via correlated inbreeding or heterozygosity, or any non-genetic effects that link parental extra-pair status to environmental variation in resource availability and/or allocation. Any combination of such downstream effects of parental extra-pair status on offspring fitness (i.e. the grandoffspring of an original polyandrous female; electronic supplementary material, figure S1) could either magnify or negate any fitness consequence of extra-pair status observed in the parental generation (i.e. the offspring of the polyandrous female; electronic supplementary material, figure S1), and thus influence the overall strength and direction of selection on female EPR.

Following from detailed consideration of the hypothesis that EPY and WPY differ in fitness [1,6,11,13,34], recent evidence suggests that phenotypic differences between maternal half-siblings can largely reflect differences in maternal and/or early environmental effects [9,10,35]. The existence of non-genetic inter-generational effects on offspring fitness has also been demonstrated more generally [17,18,20]. However, no study has tested whether an individual's fitness varies with the extra-pair status of its parents, as opposed to, or in addition to, its own extra-pair status. Such tests require fitness to be compared between offspring produced by EPY and WPY parents (rather than between EPY and WPY themselves), and consequently require paternity and fitness data encompassing three consecutive generations (see the electronic supplementary material, figure S1).

We used 18 years of comprehensive paternity and life-history data from song sparrows, *Melospiza melodia*, to compare the fitness of offspring produced by EPY versus WPY, and thereby test the hypothesis that an individual's fitness varied with the extra-pair status of its parents. We thereby consider whether inter-generational effects, either genetic or environmental, linked to extra-pair status could shape the evolution of EPR and polyandry in the wild.

## 2. Methods

### (a) Study system

A population of song sparrows resident on Mandarte Island, British Columbia, Canada has been studied intensively since 1975 [36]. All sparrows were colour-ringed as hatchlings or newly arrived immigrants, allowing subsequent identification by resighting. Both sexes can breed aged one year and females typically rear two broods per year (range 1–4) with median clutch size of four eggs (range 1–5 [36,37]). All territories were visited at least weekly each April to July to find all nests and identify all social parents (those defending territories, incubating clutches and provisioning hatchlings). All nests were visited approximately 6 days after hatching, when all hatchlings were ringed. Juveniles and adults surviving to subsequent breeding seasons were resighted with probability ≈1 [38]. Even though several nearby islands also hold song sparrows, immigration to Mandarte is infrequent (approx. 1.1 immigrants per year on average), but sufficient to maintain neutral allelic variation [39]. Local recruitment rate is high when compared with other populations and species with similar life-histories [40,41]. Repeated surveys of nearby islands have revealed few juvenile emigrants, and no adults that have bred on Mandarte have been observed elsewhere [36,41].

Since 1993, virtually all hatchlings reaching 6 days post-hatch, all adults in 1993 and all immigrants were blood-sampled [42]. All sampled individuals were genotyped at 13 microsatellite loci and assigned genetic parentage using Bayesian full probability models that incorporated genetic and spatial information [42]. Sires were assigned to 99.1 per cent of 2354 hatchlings from 1993 to 2009 with ≥95% individual level confidence [42]. Each hatchling was therefore assigned as either a WPY (sired by the female's observed social mate) or an EPY (sired by a different male) with high statistical confidence. The maximum-likelihood probability of correctly excluding a female's social mate as sire averaged 0.9998 [42]. Full details of the study system and genetic parentage analyses are provided elsewhere [36,40,42].

### (b) Analysis structure

Generalized linear mixed models (GLMMs) were used to estimate the relative fitness of hatchlings with respect to their parents' extra-pair status. Hatchling fitness was measured as (i) survival from ringing (approximately 6 days post-hatch) to recruitment (age 1 year), (ii) lifetime probability of having at least one hatched genetic offspring and (iii) lifetime probability of having at least one recruited genetic offspring (see the electronic supplementary material, figure S1). These two measures of lifetime reproductive success (LRS) were analysed as binary rather than as continuous traits (i.e. number of offspring produced) because LRS has a zero-altered distribution with low power to detect variation in non-zero LRS [43].

Two main sets of analyses were run for each of the three fitness measures. The first set included a three-level fixed effect that described whether a hatchling had two WPY parents, one EPY and one WPY parent, or two EPY parents, and hence estimated variation in hatchling fitness across these three categories of parents. The second set included two binary fixed effects that described the extra-pair status of a hatchling's father and mother, respectively, and hence estimated variation in hatchling fitness with paternal and maternal extra-pair status independently.

Given the overall extra-pair paternity rate of approximately 28 per cent, a hatchling's social father (the mother's social mate) and genetic father (sire) were the same for approximately 72 per cent of hatchlings [42]. For EPY, a hatchling's social father cannot affect its fitness through direct genetic effects. In contrast, a hatchling's genetic father could affect its fitness via genetic and/or environmental effects. All analyses were therefore run twice, considering the extra-pair status of a hatchling's social and genetic fathers, respectively. There were therefore 12 analyses in total: 3 fitness measures × 2 fixed effect structures × 2 types of father. However, analogous analyses that considered a hatchling's social and genetic fathers are not independent because only 28 per cent of hatchlings had different social and genetic fathers (see §4).

Phenotypic comparisons between EPY and WPY are typically restricted to individuals from mixed paternity broods (with at least one EPY and at least one WPY) to control for among-mother and among-brood variation in genetic and environmental effects on offspring phenotype [7,9,13]. For the same reason, we restricted our analyses to hatchlings with both parents from mixed paternity broods to minimize the possibility that variation in offspring fitness in relation to parental extra-pair status could reflect environmental covariance with the occurrence of EPR by the original grandmaternal female. Because our aim was to quantify the downstream fitness consequences of EPR for females that produce mixed paternity broods (i.e. the grandmother of the hatchlings whose fitness we compared; electronic supplementary material, figure S1), hatchlings included in analyses were not necessarily from mixed paternity broods themselves.

All analyses initially included fixed effects of a hatchling's own extra-pair status (WPY versus EPY) to account for any variation in hatchling fitness with their own extra-pair status as opposed to their parents' [40]. However, in the dataset used to quantify variation in hatchling reproductive success in relation to social father extra-pair status, all EPY hatchlings had zero offspring, preventing model convergence. These analyses were therefore run without the hatchling extra-pair status term. In all other models, estimates and conclusions remained quantitatively similar regardless of whether hatchling extra-pair status was included. Final models therefore excluded this term in order to estimate the overall relationship between parental extra-pair status and measures of hatchling fitness across hatchlings of either extra-pair status, thereby allowing direct estimation of the overall costs and benefits to the original polyandrous female. Random effects of cohort, genetic or social father identity and mother identity were included in all analyses to control for known cohort effects and repeated observations per father and mother [36,37,40]. An interaction between father and mother extra-pair status was not fitted owing to limited sample sizes (see the electronic supplementary material, table S1).

Survival from ringing to recruitment was measured for hatchlings from cohorts 1993–2009. Reproductive success (the probabilities of having at least one hatched or recruited offspring) was measured for hatchlings from cohorts 1993–2003 because some individuals from later cohorts were still alive in 2010. Four different datasets were therefore used for the twelve analyses. Analyses of recruitment used 200 hatchlings produced by 28 mothers and 25 social fathers, and 219 hatchlings produced by 34 mothers and 29 genetic fathers (see the electronic supplementary material, table S1). Analyses of reproductive success used 116 hatchlings produced by 14 mothers and 15 social fathers, and 132 hatchlings produced by 20 mothers and 21 genetic fathers. Sample sizes differ between social and genetic fathers because some hatchlings had social fathers but not genetic fathers that originated from known mixed-paternity broods (or vice versa). Data are available at the Dryad Repository: http://dx.doi.org/10.5061/dryad.6jk30.

Hatchling sex could not be included in current models owing to small available sample sizes. We therefore used chi-squared tests to test whether EPY or WPY parents produced more male than female hatchlings, or more EPY than WPY, thereby potentially confounding analyses of variation in hatchling fitness components with respect to parental extra-pair status (see electronic supplementary material, §3, tables S2 and S3).

### (c) Analysis implementation

GLMMs were fitted using Bayesian Markov chain Monte Carlo (MCMC) methods using MCMCglmm v. 2.09 in R v. 2.10.0 [44,45] specifying binary distributions and logit link functions. This approach was used to allow direct comparison with other related analyses that assumed zero altered distributions [43]. Residual variance cannot be estimated in binary models and was fixed to 1 by convention. Priors on fixed effects were normally distributed, diffuse and proper with mean zero and large variance (10^8^). Priors on variance components were inverse-Wishart distributed with parameter *V* = 1 and low degree of belief (*n* = 0.002). Prior sensitivity analysis showed that estimates of fixed effects were robust to reasonable variation in prior specifications. Furthermore, models fitted using maximum-likelihood also gave quantitatively similar results, demonstrating that conclusions were robust to fitting method. MCMC models used burn-in 50 000, 10 050 000 iterations and thinning interval 1000 to give effective sample size 10 000. First-order autocorrelation among consecutive samples was generally less than 0.05. MCMC *p*-values (the proportion of sampled parameter estimates that are less or greater than zero for positive and negative estimates, respectively) and 95% credible intervals (95% CIs) surrounding posterior means were used to summarize posterior distributions and examine whether estimated effects differed from zero. We did not correct nominal MCMC *p*-values for multiple tests because datasets and analyses are not independent and because *p*-values are Bayesian rather than frequentist. Rather, we use means, 95%CIs and *p*-values as tools to describe and interpret posterior distributions. To provide clear visualization of estimated effects, posterior means and 95% CIs were back-transformed onto observed data scales, marginalizing over random effects.

## 3. Results

Across the datasets describing variation in hatchling fitness with respect to social and genetic father extra-pair status, respectively, 14 per cent (28/200) and 15 per cent (33/219) of hatchlings survived to recruit, 12 per cent (14/116) and 14 per cent (19/132) had at least one hatched offspring, and 9 per cent (10/116) and 10 per cent (13/132) had at least one recruited offspring. Offspring extra-pair status and sex were not closely associated with parental extra-pair status across these datasets (see the electronic supplementary material, tables S2 and S3).

### (a) Recruitment: mother and social father status

**Figure 1.**
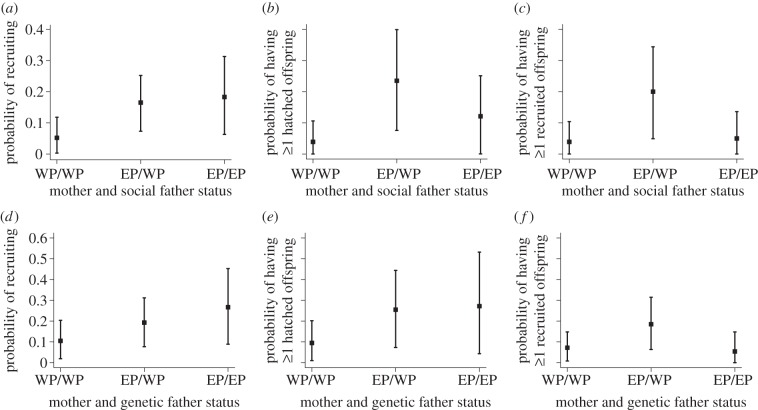
Back-transformed estimates (with 95% credible intervals) of a hatchling's probability of (*a*,*d*) surviving to recruit, (*b*,*e*) having at least one hatched offspring and (*c*,*f*) having at least one recruited offspring. WP/WP, EP/WP and EP/EP indicate hatchlings with two WPY parents, one EPY and one WPY parent, and two EPY parents, respectively, relative to their (*a–c*) social father and (*d–f*) genetic father extra-pair status. Values correspond to models run without a term describing a hatchling's own extra-pair status and therefore show estimates averaged over EPY and WPY hatchlings.

Modelling parental extra-pair status as a three-level fixed effect showed that EPY hatchlings were less likely to recruit than WPY hatchlings, and that hatchlings with one or two EPY parents were marginally more likely to recruit than hatchlings with two WPY parents (figure 1*a* and table 1).

**Table 1.** Generalized linear mixed models explaining variation in a hatchling's probability of survival to recruitment, and lifetime probability of having at least one hatched offspring and at least one recruited offspring with respect to mother extra-pair status and (*a*) social father extra-pair status or (*b*) genetic father extra-pair status. Models were first run (i) including a three-level effect of parental extra-pair status describing whether none (intercept), one (WP/EP) or both parents (EP/EP) were EPY. Models were then run (ii) including two two-level effects of mother and father extra-pair status where the intercept corresponds to hatchlings with a WPY mother and a WPY father. A main effect of the hatchling's own extra-pair status was also modelled where feasible (EPY status; see §2). Estimates for hatchling extra-pair status are from models including this term and correspond to EPY; all other estimates are from models excluding this term. Mean estimates with 95% CI and MCMC *p*-values are presented. Bold indicates nominally statistically significant terms with MCMC *p* ≤ 0.05. Estimated effects are visualized in figures 1 and 2.

				model structure							
				(i) three-level parental status				(ii) binary parental status of mother and father			
model		sample size	parameter	intercept	WP/EP	EP/EP	EPY status	intercept	mother	father	EPY status
survival to recruitment	(a) social father	200	mean	**−3.95**	**1.85**	**1.99**	**−1.92**	**−3.78**	0.01	**1.85**	0.75
			95% CI	**−5.90, −2.23**	**−0.14, 3.80**	−**0.09, 4.23**	−**3.74,** −**0.06**	−**5.33,** −**2.27**	−1.25, 1.30	**0.34, 3.49**	−0.99, 2.49
			MCMC *p*	**<0.01**	**0.05**	**0.05**	**0.02**	**<0.01**	0.99	**0.02**	0.36
	(b) genetic father	219	mean	−**3.43**	1.19	1.81	−0.15	−**5.37**	−0.06	**1.82**	−0.08
			95% CI	−**5.30,** −**1.86**	−0.56, 2.89	−0.29, 4.03	−1.58, 1.27	−**8.75,** −**2.11**	−1.54, 1.39	**0.35, 3.33**	−1.46, 1.30
			MCMC *p*	**<0.01**	0.17	0.08	0.83	**<0.01**	0.92	**0.02**	0.91
hatched offspring	(a) social father	116	mean	−**5.00**	**3.29**	2.02	—	−**4.99**	−1.26	**3.28**	—
			95% CI	−**8.23,** −**2.37**	**0.52, 6.64**	−1.18, 5.84	—	−**8.13,** −**2.33**	−3.68, 1.05	**0.55, 6.61**	—
			MCMC *p*	**<0.01**	**0.01**	0.21	—	**<0.01**	0.24	**0.01**	—
	(b) genetic father	132	mean	−**4.35**	**2.30**	2.40	−0.03	−**4.25**	−0.19	**2.38**	0.17
			95% CI	−**7.10,** −**2.12**	**0.18, 4.46**	−0.69, 5.62	−1.82, 1.74	−**6.80,** −**2.16**	−3.00, 2.69	**0.43, 4.42**	−1.63, 1.90
			MCMC *p*	**<0.01**	**0.03**	0.10	0.99	**<0.01**	0.83	**0.02**	0.83
recruited offspring	(a) social father	116	mean	−**5.09**	**3.06**	0.388	—	−**5.07**	−2.66	**3.03**	—
			95% CI	−**8.39,** −**2.41**	**0.19, 6.44**	−4.06, 4.49	—	−**8.39,** −**2.37**	−6.17, 0.48	**0.16, 6.42**	—
			MCMC *p*	**<0.01**	**0.02**	0.84	—	**<0.01**	0.07	**0.03**	—
	(b) genetic father	132	mean	**−3.57**	1.54	−0.75	−0.30	−**3.57**	−2.55	**1.71**	−0.02
			95% CI	−**5.28, 1.98**	−0.12, 3.39	−4.39, 2.22	−2.23, 1.39	−**5.28,** −**2.06**	−5.65, 0.32	**0.08, 3.52**	−1.82, 1.81
			MCMC *p*	**<0.01**	0.07	0.65	0.77	**<0.01**	0.06	**0.04**	0.99

**Figure 2.**
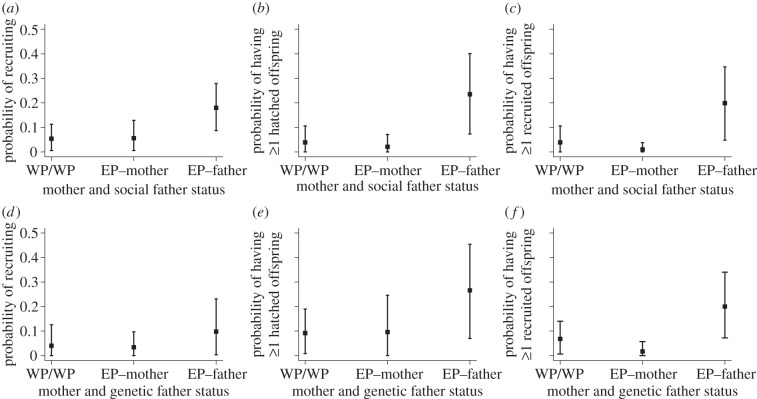
Back-transformed estimates (with 95% credible intervals) of a hatchling's probability of (*a*,*d*) surviving to recruit, (*b*,*e*) having at least one hatched offspring and (*c*,*f*) having at least one recruited offspring. WP/WP indicates hatchlings whose mother and father were both WPY, EP–mother indicates hatchlings with an EPY mother and a WPY father, and EP–father indicates hatchlings with an EPY father and a WPY mother relative to (*a–c*) social father and (*d–f*) genetic father extra-pair status. Figures correspond to models run without a term describing a hatchling's own extra-pair status, and therefore show estimates averaged over EPY and WPY hatchlings.

Models with parental extra-pair status as binary fixed effects showed no effect of hatchling extra-pair status. However, hatchlings with an EPY social father were more likely to recruit than hatchlings with a WPY social father while hatchlings with an EPY mother were similarly likely to recruit as hatchlings with a WPY mother (figure 2*a* and table 1).

### (b) Recruitment: mother and genetic father status

Models with parental extra-pair status as a three-level fixed effect showed no significant effect of hatchling extra-pair status on recruitment. However, hatchlings with two EPY parents were marginally more likely to recruit than hatchlings with two WPY parents, while hatchlings with one EPY parent also tended to be so (figure 1*d* and table 1).

Models with parental status as binary fixed effects showed that recruitment did not vary significantly with a hatchling's own extra-pair status or its mother's (figure 2*d* and table 1). However, hatchlings with an EPY genetic father were more likely to recruit than hatchlings with a WPY genetic father (figure 2*d* and table 1).

### (c) Hatched offspring: mother and social father status

Modelling parental extra-pair status as a three-level fixed effect showed that hatchlings with one EPY parent were more likely to have at least one hatched offspring than hatchlings with two WPY parents, while hatchlings with two EPY parents tended to have at least one offspring (figure 1*b* and table 1).

Models with parental status as binary fixed effects showed that hatchlings with an EPY social father were substantially more likely to have at least one hatched offspring than hatchlings with a WPY social father (figure 2*b* and table 1). Hatchlings with an EPY mother, if anything, tended to be less likely to have at least one hatched offspring than hatchlings with a WPY mother (figure 2*b* and table 1).

### (d) Hatched offspring: mother and genetic father status

Modelling parental extra-pair status as a three-level fixed effect showed no significant effect of a hatchling's own extra-pair status but that hatchlings with one EPY parent were more likely to have at least one hatched offspring than hatchlings with two WPY parents (figure 1*e* and table 1). Hatchlings with two EPY parents tended to be more likely to have at least one hatched offspring than hatchlings with two WPY parents (figure 1*e* and table 1).

Models with parental status as binary fixed effects showed no significant effect of a hatchling's own extra-pair status or its mother's (figure 2*e* and table 1). However, hatchlings with an EPY genetic father were more likely to have at least one hatched offspring than hatchlings with a WPY genetic father (figure 2*e* and table 1).

### (e) Recruited offspring: mother and social father status

Modelling parental extra-pair status as a three-level fixed effect showed that hatchlings with one EPY parent were more likely to have at least one recruited offspring than hatchlings with two WPY parents (figure 1*c* and table 1). Hatchlings with two EPY parents were similarly likely to have at least one recruited offspring as hatchlings with two WPY parents (figure 1*c* and table 1).

Models with parental status as binary fixed effects showed that hatchlings with an EPY social father were substantially more likely to have at least one recruited offspring than hatchlings with a WPY social father (figure 2*c* and table 1). In contrast, hatchlings with an EPY mother tended to be less likely to have at least one recruited offspring than hatchlings with a WPY mother (figure 2*c* and table 1).

### (f) Recruited offspring: mother and genetic father status

Modelling parental extra-pair status as a three-level fixed effect showed no significant effects of a hatchling's own extra-pair status or its parents' (figure 1*f* and table 1). However, hatchlings with one EPY parent tended to be more likely to have at least one recruited offspring than hatchlings with two WPY parents or with two EPY parents (figure 1*f* and table 1).

Models with parental status as binary fixed effects showed that hatchlings with an EPY genetic father were more likely to have at least one recruited offspring than hatchlings with a WPY genetic father (figure 2*f* and table 1). Hatchlings with an EPY mother were marginally less likely to have at least one recruited offspring than hatchlings with a WPY mother (figure 2*f* and table 1).

## 4. Discussion

Multiple inter-generational effects, potentially comprising interacting genetic and environmental effects, can shape offspring phenotype and influence evolutionary and phenotypic dynamics [16,17,19–21,27]. However, despite huge interest in the evolution of EPR and polyandry, inter-generational relationships between parental extra-pair status and offspring fitness have not been quantified in wild populations. A single laboratory study compared fitness between grandoffspring of polyandrous and monandrous females, and found that grandoffspring of polyandrous females were more likely to reach adulthood in red flour beetles (*Tribolium castaneum*) under some conditions [46]. Rather, numerous studies have tested for effects of an individual's own extra-pair status on its own fitness [1,12,13]. We compared survival to recruitment and the lifetime probability of having at least one hatched or recruited offspring between offspring of EPY and WPY parents originating from mixed paternity broods, and demonstrate substantial variation in offspring fitness in relation to parental extra-pair status.

### (a) Parental extra-pair status and offspring fitness

The requirement for three generations of comprehensive paternity data meant that even given our 18-year dataset, sample sizes available to relate offspring lifetime fitness to the extra-pair status of parents from known mixed-paternity broods were inevitably restricted. However, estimated effects were, in many cases, large and nominally statistically significant despite relatively low power. Overall, hatchlings with two EPY parents tended to be more likely to recruit and to produce at least one hatched offspring over their lifetimes than hatchlings with two WPY parents. Furthermore, hatchlings with one EPY and one WPY parent were more likely to recruit and to have at least one offspring than hatchlings with two WPY parents, and also tended to be more likely to have at least one offspring than hatchlings with two EPY parents. However, most hatchlings in our dataset with one EPY and one WPY parent had a WPY mother and EPY father rather than the reverse, reflecting the low average survival of female EPY in the study population [40]. The first analyses therefore cannot definitively distinguish effects of having one EPY parent versus specifically having an EPY father. However, further analyses that treated mother and father extra-pair status as independent effects revealed somewhat different relationships between hatchling fitness and parental status. Specifically, hatchlings with EPY fathers were substantially more likely to recruit and to have at least one offspring than hatchlings with WPY fathers. By contrast, hatchlings with EPY mothers were similarly likely to recruit and, if anything, less likely to have at least one offspring than hatchlings with WPY mothers.

Relationships between an individual's fitness and its own extra-pair status and that of its parents would be confounded if EPY and WPY parents consistently produced EPY and WPY offspring, respectively. However, hatchling extra-pair status was far from completely confounded with parent extra-pair status (see the electronic supplementary material, tables S2 and S3), and most models explicitly controlled for hatchling status. Furthermore, there was no marked sex bias in hatchlings produced by EPY versus WPY parents (see the electronic supplementary material, tables S2 and S3). Our results therefore suggest that major components of offspring fitness vary in contrasting directions in relation to the extra-pair status of their mother and father independent of the offspring's own extra-pair status.

Variation in offspring fitness with respect to parental extra-pair status could potentially increase or decrease the total benefit or cost of EPR to an original polyandrous female (see the electronic supplementary material, figure S1). This depends on whether variation in offspring fitness with parental extra-pair status reinforces or opposes variation in fitness with an individual's own extra-pair status, and on the causes of these patterns. Previous analyses of song sparrow data showed that, contrary to widespread prediction, EPY tend to be less likely to recruit and to have at least one offspring than their maternal half-sibling WPY on average [40,43]. In contrast, current analyses show that major fitness components were higher in hatchlings with EPY parents than hatchlings with WPY parents, and estimated effects were often large. For example, hatchlings with at least one EPY parent were up to 22 to 83 per cent more likely to recruit and to have at least one hatched offspring relative to hatchlings with two WPY parents. The overall pattern of variation in hatchling fitness in relation to parental extra-pair status therefore opposed the pattern of fitness variation among EPY and WPY themselves. Such opposing variation in fitness in relation to an offspring's own extra-pair status versus its parents' status would probably decrease any total benefit or cost of EPR to the original polyandrous female.

However, the situation is further complicated by the observation that components of hatchling fitness varied in different ways relative to their mother's versus their father's extra-pair status. For example, hatchlings with EPY mothers were up to 75 per cent less likely to have at least one offspring than hatchlings with WPY mothers. In contrast, hatchlings with EPY fathers were 59 to 83 per cent more likely to recruit or have at least one offspring than hatchlings with WPY fathers. Interestingly, this sex-specific variation broadly matches observed variation in hatchling recruitment with respect to a hatchling's own extra-pair status; female EPY were less likely to recruit and have at least one offspring than female WPY, whereas male EPY tended to be more likely to recruit and to have at least one offspring than male WPY [40]. Variation in components of hatchling fitness with respect to parental extra-pair status therefore tended to reinforce variation in fitness with respect to an individual's own extra-pair status when parental sex was considered. Such parallel variation in the fitness of a polyandrous female's offspring and grandoffspring may therefore intensify selection on female EPR.

In fact, when these inter-generational patterns are combined, the net result is that EPY tend to leave fewer grandoffspring than their maternal half-sib WPY, suggesting that EPR may be costly to polyandrous females [43]. This overall effect was primarily due to low fitness of EPY females which, as our current analyses show, is partly due to the low fitness of the offspring of these EPY females.

### (b) Mechanisms

The evolutionary consequences of inter-generational associations between parent extra-pair status and offspring fitness will depend on the causal genetic and environmental mechanisms [14,16–20]. Numerous interacting mechanisms could be involved, and there is as yet no clear theory predicting the directions or magnitudes of sex-specific effects that might be expected under any specific conditions.

First, parallel variation in hatchling fitness with their own extra-pair status and that of their parents could result from additive genetic variation (and hence heritability) in fitness [15,21,47,48]. For example, if EPR systematically resulted in EPY of higher or lower additive genetic value than their WPY maternal half-siblings (as widely hypothesized [1,6,12,34]), EPY would have offspring of relatively high or low additive genetic value on average. Indeed, recruitment shows non-zero heritability in song sparrows, and EPY of both sexes have lower additive genetic value for recruitment than the WPY they replaced [11]. Additive genetic effects are therefore likely to cause some association between offspring recruitment and parental extra-pair status.

However, offspring with EPY fathers were more likely to recruit than offspring with WPY fathers even though EPY have a lower genetic value for recruitment than WPY, on average [11]. The observed relationships between offspring fitness and parental extra-pair status therefore cannot simply or solely reflect additive genetic effects. Furthermore, hatchling fitness varied with the extra-pair status of both social and genetic fathers. Since a social father can affect the fitness of offspring he rears via environmental effects, but not via direct genetic effects, this suggests that at least some of the inter-generational effect of parental extra-pair status is environmental. On the other hand, because only 28 per cent of hatchlings had different genetic and social fathers overall, effects of social versus genetic sires cannot be definitively separated through phenotypic analyses.

Second, an association between parental extra-pair status and hatchling fitness could reflect more complicated genetic effects that can arise in small populations, including our song sparrow population. For example, EPR is often hypothesized to allow inbreeding avoidance, thereby causing EPY and WPY to differ in *f* [25,34,49]. A difference in *f* between EPY and WPY might then be reflected in their offspring via positive correlations between parent and offspring *f* that can arise in small populations with variance in relatedness [26,27]. Given inbreeding depression in fitness, which occurs in song sparrows and other species [50–54], a systematic difference in *f* between EPY and WPY might therefore be translated into a correlated difference in *f* and fitness between their offspring. However, to explain our current results, polyandrous females would need to avoid inbreeding through sons but not daughters; this remains to be tested.

Third, differences in fitness between offspring of EPY and WPY could reflect direct or indirect environmental effects. For example, variation in resource availability or allocation between EPY and WPY may affect parental investment in offspring or the conditions these offspring experience [14,18]. Inter-generational parental and environmental effects have been demonstrated in numerous other contexts and systems [28–31,33], and can be sex-specific [32]. Such environmental effects could arise whether phenotypic differences in fitness between the EPY and WPY parents are genetic or environmental, or both [16]. For example, a difference in *f* between EPY and WPY half-siblings could cause inter-generational effects on offspring fitness if more inbred females invest less in their offspring [55–57]. Indeed, phenotypic differences between EPY and WPY can at least partly reflect differences in natal environment or maternal investment between half-siblings [9,10,35,58].

We cannot yet distinguish these various mechanisms. However, our results show that future studies aiming to quantify the overall selection on EPR may need to estimate both the fitness of a polyandrous female's WPY and EPY sons and daughters in the first generation, and the fitness of grandoffspring produced by those WPY and EPY in the second generation. They also further indicate that phenotypic differences between maternal half-sib EPY and WPY may be environmental rather than solely genetic, and demonstrate the utility of inter-generational analyses in probing such effects. Quantifying this inter-generational variation and distinguishing the underlying genetic and environmental mechanisms will be challenging, but may be the key to understanding the evolution and persistence of genetic polyandry in socially monogamous populations.

## References

[1] Arnqvist G. Kirkpatrick M. 2005 The evolution of infidelity in socially monogamous passerines: the strength of direct and indirect selection on extrapair copulation behavior in females. Am. Nat. 165, S26–S37 10.1086/429350 (doi:10.1086/429350) 15795859

[2] Westneat D. F. Stewart I. R. K. 2003 Extra-pair paternity in birds: causes, correlates, and conflict. Annu. Rev. Ecol. Evol. Syst. 34, 365–396 10.1046/j.1365–294X.2002.01613.x (doi:10.1046/j.1365–294X.2002.01613.x)

[3] Albrecht T. Kreisinger J. Pialek J. 2006 The strength of direct selection against female promiscuity is associated with rates of extrapair fertilizations in socially monogamous songbirds. Am. Nat. 167, 739–744 10.1086/502633 (doi:10.1086/502633) 16671017

[4] Griffith S. C. 2007 The evolution of infidelity in socially monogamous passerines: neglected components of direct and indirect selection. Am. Nat. 169, 274–281 10.1086/510601 (doi:10.1086/510601) 17211810

[5] Forstmeier W. Martin K. Bolund E. Schielzeth H. Kempenaers B. 2011 Female extrapair mating behavior can evolve via indirect selection on males. Proc. Natl Acad. Sci. USA 108, 10 608–10 613 10.1073/pnas.1103195108 (doi:10.1073/pnas.1103195108) PMC3127899 21670288

[6] Griffith S. C. Owens I. P. F. Thuman K. A. 2002 Extra pair paternity in birds: a review of interspecific variation and adaptive function. Mol. Ecol. 11, 2195–2212 10.1046/j.1365-294X.2002.01613.x (doi:10.1046/j.1365-294X.2002.01613.x) 12406233

[7] Sheldon B. C. Merila J. Qvarnstrom A. Gustafsson L. Ellegren H. 1997 Paternal genetic contribution to offspring condition predicted by size of male secondary sexual character. Proc. R. Soc. Lond. B 264, 297–302 10.1098/rspb.1997.0042 (doi:10.1098/rspb.1997.0042)

[8] Kempenaers B. Verheyren G. R. Dhondt A. A. 1997 Extrapair paternity in the blue tit *Parus* (*caeruleus*): female choice, male characteristics, and offspring quality. Behav. Ecol. 8, 481–492 10.1093/beheco/8.5.481 (doi:10.1093/beheco/8.5.481)

[9] Magrath M. J. L. Vedder O. van der Velde M. Komdeur J. 2009 Maternal effects contribute to the superior performance of extra-pair offspring. Curr. Biol. 19, 792–797 10.1016/j.cub.2009.03.068 (doi:10.1016/j.cub.2009.03.068) 19409787

[10] Ferree E. D. Dickinson J. Rendell W. Stern C. Porter S. 2010 Hatching order explains an extrapair chick advantage in western bluebirds. Behav. Ecol. 21, 802–807 10.1093/beheco/arq056 (doi:10.1093/beheco/arq056)

[11] Reid J. M. Sardell R. J. 2012 Indirect selection on female extra-pair reproduction? Comparing the additive genetic value of maternal half-sib extra-pair and within-pair offspring. Proc. R. Soc. B 279, 1700–1708 10.1098/rspb.2011.2230 (doi:10.1098/rspb.2011.2230) PMC3297462 22113036

[12] Akçay E. Roughgarden J. 2007 Extra-pair paternity in birds: review of the genetic benefits. Evol. Ecol. Res. 9, 855–868

[13] Schmoll T. Schurr F. M. Winkel W. Epplen J. T. Lubjuhn T. 2009 Lifespan, lifetime reproductive performance and paternity loss of within-pair and extra-pair offspring in the coal tit *Periparus ater*. Proc. R. Soc. B 276, 337–345 10.1098/rspb.2008.1116 (doi:10.1098/rspb.2008.1116) PMC2674355 18812289

[14] Mousseau T. A. Fox C. W. 1998 The adaptive significance of maternal effects. Trends Ecol. Evol. 13, 403–407 10.1016/S0169-5347(98)01472-4 (doi:10.1016/S0169-5347(98)01472-4) 21238360

[15] Merila J. Sheldon B. C. 2000 Lifetime reproductive success and heritability in nature. Am. Nat. 155, 301–310 10.1086/303330 (doi:10.1086/303330) 10718727

[16] Hunt J. Simmons L. W. 2002 The genetics of maternal care: direct and indirect genetic effects on phenotype in the dung beetle *Onthophagus taurus*. Proc. Natl Acad. Sci. USA 99, 6828–6832 10.1073/pnas.092676199 (doi:10.1073/pnas.092676199) 11983863 PMC124488

[17] Bonduriansky R. Day T. 2009 Nongenetic inheritance and its evolutionary implications. Ann. Rev. Ecol. Evol. Syst. 40, 103–125 10.1146/annurev.ecolsys.39.110707.173441 (doi:10.1146/annurev.ecolsys.39.110707.173441)

[18] Monaghan P. 2008 Early growth conditions, phenotypic development and environmental change. Proc. R. Soc. B 363, 1635–1645 10.1098/rstb.2007.0011 PMC2606729 18048301

[19] Day T. Bonduriansky R. 2011 A unified approach to the evolutionary consequences of genetic and nongenetic inheritance. Am. Nat. 178, E18–E36 10.1086/660911 (doi:10.1086/660911) 21750377

[20] Uller T. 2008 Developmental plasticity and the evolution of parental effects. Trends Ecol. Evol. 23, 432–438 10.1016/j.tree.2008.04.005 (doi:10.1016/j.tree.2008.04.005) 18586350

[21] Kruuk L. E. B. Clutton-Brock T. H. Slate J. Pemberton J. M. Brotherstone S. Guinness F. E. 2000 Heritability of fitness in a wild mammal population. Proc. Natl Acad. Sci. USA 97, 698–703 10.1073/pnas.97.2.698 (doi:10.1073/pnas.97.2.698) 10639142 PMC15393

[22] Lynch M. Walsh B. 1998 Genetics and analysis of quantitative traits. Sunderland, MA: Sinauer

[23] Mitton J. B. Schuster W. S. Cothran E. G. De Fries J. C. 1993 Correlation between the individual heterozygosity of parents and their offspring. Heredity 71, 59–63 10.1038/hdy.1993.107 (doi:10.1038/hdy.1993.107) 8360078

[24] Garcia-Navas V. Ortego J. Sanz J. 2009 Heterozygosity-based assortative matingin blue tits (*Cyanistes caeruleus*): implications for the evolution of mate choice. Proc. R. Soc. B 276, 2931–2940 10.1098/rspb.2009.0417 (doi:10.1098/rspb.2009.0417) PMC2817209 19474042

[25] Kempenaers B. 2007 Mate choice and genetic quality: A review of the heterozygosity theory. Adv. Stud. Behav. 37, 189–278 10.1016/S0065-3454(07)37005-8 (doi:10.1016/S0065-3454(07)37005-8)

[26] Reid J. M. Arcese P. Keller L. F. 2006 Intrinsic parent–offspring correlation in inbreeding level in a song sparrow (*Melospiza melodia*) population open to immigration. Am. Nat. 168, 1–13 10.1086/504852 (doi:10.1086/504852) 16685634

[27] Reid J. M. Keller L. F. 2010 Correlated inbreeding among relatives: occurrence, magnitude, and implications. Evolution 64, 973–985 10.1111/j.1558-5646.2009.00865.x (doi:10.1111/j.1558-5646.2009.00865.x) 19817848

[28] Plaistow S. J. Lapsley C. T. Benton T. G. 2006 Context-dependent intergenerational effects: the interaction between past and present environments and its effect on population dynamics. Am. Nat. 167, 206–215 10.1086/499380 (doi:10.1086/499380) 16670981

[29] Van De Pol M. Bruinzeel L. W. Heg D. Van Der Jeugd H. P. Verhulst S. 2006 A silver spoon for a golden future: long-term effects of natal origin on fitness prospects of oystercatchers (*Haematopus ostralegus*). J. Anim. Ecol. 75, 616–626 10.1111/j.1365-2656.2006.01079.x (doi:10.1111/j.1365-2656.2006.01079.x) 16638014

[30] Naguib M. Nemitz A. Gil D. 2006 Maternal developmental stress reduces reproductive success of female offspring in zebra finches. Proc. R. Soc. B 273, 1901–1905 10.1098/rspb.2006.3526 (doi:10.1098/rspb.2006.3526) PMC1634771 16822750

[31] Gilbert L. Williamson K. A. Graves J. A. 2011 Male attractiveness regulates daughter fecundity non-genetically via maternal investment. Proc. R. Soc. B 279, 523–528 10.1098/rspb.2011.0962 (doi:10.1098/rspb.2011.0962) PMC3234556 21733898

[32] Bonduriansky R. Head M. 2007 Maternal and paternal condition effects on offspring phenotype in *Telostylinus angusticollis* (Diptera: Neriidae). J. Evol. Biol. 20, 2379–2388 10.1111/j.1420-9101.2007.01419.x (doi:10.1111/j.1420-9101.2007.01419.x) 17956399

[33] Kerr T. D. Boutin D. LaMontagne J. J. McAdam A. G. Humphries M. M. 2007 Persistent maternal effects on juvenile survival in North American red squirrels. Biol. Lett. 3, 289–291 10.1098/rsbl.2006.0615 (doi:10.1098/rsbl.2006.0615) 17395572 PMC2464683

[34] Jennions M. D. Petrie M. 2000 Why do females mate multiply? A review of the genetic benefits. Biol. Rev. 75, 21–64 10.1017/S0006323199005423 (doi:10.1017/S0006323199005423) 10740892

[35] Tschirren B. Postma E. Rutstein A. N. Griffith S. C. 2012 When mothers make sons sexy: maternal effects contribute to the increased sexual attractiveness of extra-pair offspring. Proc. R. Soc. B 279, 1233–1240 10.1098/rspb.2011.1543 (doi:10.1098/rspb.2011.1543) PMC3267142 21957136

[36] Smith J. N. M. Keller L. F. Marr A. B. Arcese P. 2006 Conservation and biology of small populations: the song sparrows of Mandarte Island. New York, NY: Oxford University Press

[37] Keller L. F. Reid J. M. Arcese P. 2008 Testing evolutionary models of senescence in a natural population: age and inbreeding effects on fitness components in song sparrows. Proc. R. Soc. B 275, 597–604 10.1098/rspb.2007.0961 (doi:10.1098/rspb.2007.0961) PMC2366077 18211879

[38] Wilson S. Norris D. R. Wilson A. G. Arcese P. 2007 Breeding experience and population density affect the ability of a songbird to respond to future climate variation. Proc. R. Soc. B 274, 2539–2545 10.1098/rspb.2007.0643 (doi:10.1098/rspb.2007.0643) PMC2275880 17698488

[39] Keller L. F. Jeffery K. J. Arcese P. Beaumont M. A. Hochachka W. M. Smith J. N. M. Bruford M. W. 2001 Immigration and the ephemerality of a natural population bottleneck: evidence from molecular markers. Proc. R. Soc. Lond. B 268, 1387–1394 10.1098/rspb.2001.1607 (doi:10.1098/rspb.2001.1607) PMC1088753 11429139

[40] Sardell R. J. Arcese P. Keller L. F. Reid J. M. 2011 Sex-specific differential survival of extra-pair and within-pair offspring in song sparrows (*Melospiza melodia*). Proc. R. Soc. B 278, 3251–3259 10.1098/rspb.2011.0173 (doi:10.1098/rspb.2011.0173) PMC3169025 21389032

[41] Wilson A. G. Arcese P. 2008 Influential factors for natal dispersal in an avian island metapopulation. J. Avian Biol. 39, 341–347 10.1111/j.2008.0908-8857.04239.xER (doi:10.1111/j.2008.0908-8857.04239.xER)

[42] Sardell R. J. Keller L. F. Arcese P. Bucher T. Reid J. M. 2010 Comprehensive paternity assignment; genotype, spatial location and social status in song sparrows, *Melospiza melodia*. Mol. Ecol. 19, 4352–4364 10.1111/j.1365-294X.2010.04805.x (doi:10.1111/j.1365-294X.2010.04805.x) 20819155

[43] Sardell R. J. Arcese P. Keller L. F. Reid J. M. 2012 Are there indirect fitness benefits of female extra-pair reproduction? Lifetime reproductive success of within-pair and extra-pair offspring. Am. Nat. 179, 779–793 10.1086/665665 (doi:10.1086/665665) 22617265

[44] R Development Core Team 2009 R: a language and environment for statistical computing. Vienna, Austria: R Foundation for Statistical Computing.

[45] Hadfield J. D. 2010 MCMC methods for multi-response generalized linear mixed models: the MCMCglmm R package. J. Stat. Softw. 33, 1–2220808728

[46] Pai A. Yan G. 2002 Polyandry produces sexy sons at the cost of daughters in red flour beetles. Proc. R. Soc. Lond. B 269, 361–368 10.1098/rspb.2001.1893 (doi:10.1098/rspb.2001.1893) PMC1690898 11886623

[47] McCleery R. H. Pettifor R. A. Armbruster P. Meyer K. Sheldon B. C. Perrins C. M. 2004 Components of variance underlying fitness in a natural population of the great tit *Parus major*. Am. Nat. 164, E62–E72 10.1086/422660 (doi:10.1086/422660) 15478083

[48] Coltman D. W. O'Donoghue P. Hogg J. T. Festa-Bianchet M. 2005 Selection and genetic (co)variance in bighorn sheep. Evolution 59, 1372–1382 16050112

[49] Tregenza T. Wedell N. 2000 Genetic compatibility, mate choice and patterns of parentage: invited review. Mol. Ecol. 9, 1013–1027 10.1046/j.1365-294x.2000.00964.x (doi:10.1046/j.1365-294x.2000.00964.x) 10964221

[50] Keller L. F. 1998 Inbreeding and its fitness effects in an insular population of song sparrows (*Melospiza melodia*). Evolution 52, 240–250 10.2307/2410939 (doi:10.2307/2410939) 28568167

[51] Keller L. F. Waller D. M. 2002 Inbreeding effects in wild populations. Trends Ecol. Evol. 17, 230–241 10.1016/S0169-5347(02)02489-8 (doi:10.1016/S0169-5347(02)02489-8)

[52] Jensen H. Bremset E. M. Ringsby T. H. Saether B. 2007 Multilocus heterozygosity and inbreeding depression in an insular house sparrow metapopulation. Mol. Ecol. 16, 4066–4078 10.1111/j.1365-294X.2007.03452.x (doi:10.1111/j.1365-294X.2007.03452.x) 17894759

[53] Szulkin M. Garant D. McCleery R. H. Sheldon B. C. 2007 Inbreeding depression along a life-history continuum in the great tit. J. Evol. Biol. 20, 1531–1543 10.1111/j.1420-9101.2007.01325.x (doi:10.1111/j.1420-9101.2007.01325.x) 17584246

[54] Simmons L. W. 2011 Inbreeding depression in the competitive fertilization success of male crickets. J. Evol. Biol. 24, 415–421 10.1111/j.1420-9101.2010.02179.x (doi:10.1111/j.1420-9101.2010.02179.x) 21091574

[55] Reid J. M. Arcese P. Keller L. F. 2003 Inbreeding depresses immune response in song sparrows (*Melospiza melodia*): direct and inter-generational effects. Proc. R. Soc. Lond. B 270, 2151–2157 10.1098/rspb.2003.2480 (doi:10.1098/rspb.2003.2480) PMC1691491 14561279

[56] Richardson D. S. Komdeur J. Burke T. 2004 Inbreeding in the Seychelles warbler: environment-dependent maternal effects. Evolution 58, 2037–2048 15521460 10.1111/j.0014-3820.2004.tb00488.x

[57] Marr A. B. Arcese P. Hochachka W. M. Reid J. M. Keller L. F. 2006 Interactive effects of environmental stress and inbreeding on reproductive traits in a wild bird population. J. Anim. Ecol. 75, 1406–1415 10.1111/j.1365-2656.2006.01165.x (doi:10.1111/j.1365-2656.2006.01165.x) 17032373

[58] Johnson L. S. Brubaker J. L. Johnson B. G. P. Masters B. S. 2009 Evidence for a maternal effect benefiting extra-pair offspring in a songbird, the house wren *Troglodytes aedon*. J. Avian Biol. 40, 248–253 10.1111/j.1600-048X.2009.04777.x (doi:10.1111/j.1600-048X.2009.04777.x)

